# Determination of genetic associations between indels in 11 candidate genes and milk composition traits in Chinese Holstein population

**DOI:** 10.1186/s12863-019-0751-y

**Published:** 2019-05-28

**Authors:** Jianping Jiang, Lin Liu, Yahui Gao, Lijun Shi, Yanhua Li, Weijun Liang, Dongxiao Sun

**Affiliations:** 1 Department of Animal Genetics and Breeding, College of Animal Science and Technology, Key Laboratory of Animal Genetics and Breeding of Ministry of Agriculture, National Engineering Laboratory for Animal Breeding, China Agricultural University, 2 Yuanmingyuan West Road, Beijing, 100193 China; 2 Beijing Dairy Cattle Center, Beijing, 100085 China; 3 College of Animal Science and Technology, Guangxi University, Nanning, 530004 China

**Keywords:** Indel, Candidate gene, Genetic effect, Milk composition traits, Dairy cattle

## Abstract

**Background:**

We have previously identified 11 promising candidate genes for milk composition traits by resequencing the whole genomes of 8 Holstein bulls with extremely high and low estimated breeding values for milk protein and fat percentages (high and low groups), including *FCGR2B*, *CENPE*, *RETSAT*, *ACSBG2*, *NFKB2*, *TBC1D1*, *NLK*, *MAP3K1*, *SLC30A2*, *ANGPT1* and *UGDH* those contained 25 indels between high and low groups. In this study, the purpose was to further examine whether these candidates have significant genetic effects on milk protein and fat traits.

**Results:**

With PCR product sequencing, 13 indels identified by whole genome resequencing were successfully genotyped. With association analysis in 769 Chinese Holstein cows, we found that the indel in *FCGR2B* was significantly associated with milk yield, protein yield and protein percentage (*P* = 0.0041 to 0.0297); five indels in *CENPE* and one indel in *MAP3K1* were markedly relevant to milk yield, fat yield and protein yield (*P* < 0.0001 to 0.0073); polymorphism in *RETSAT* was evidently associated with milk yield, fat yield, protein yield and protein percentage (*P* = 0.0001 to 0.0237); variant in *ACSBG2* affected fat yield and protein percentage (*P* = 0.0088 and 0.0052); one indel in *TBC1D1* was with respect to fat percentage and protein percentage (*P *= 0.0224 and 0.0209). Significant associations were shown between indels in *NLK* and protein yield and protein percentage (*P* = 0.0012 to 0.0257); variant in *UGDH* was related to the milk yield (*P* = 0.0312). The two exonic indels in *FCGR2B* and *CENPE* were predicted to change the mRNA and protein secondary structures, and resulted in the corresponding protein dysfunction.

**Conclusion:**

Our findings presented here provide the first evidence for the associations of eight functional genes with milk yield and composition traits in dairy cattle.

**Electronic supplementary material:**

The online version of this article (10.1186/s12863-019-0751-y) contains supplementary material, which is available to authorized users.

## Backgroud

In dairy cattle, milk yield and milk composition traits are the most important economic traits, which are controlled by numerous environmental factors and genes [1–4]. Over the past decades, unraveling the major genes and causal mutations with large effect on milk yield and composition traits is one of the important research fields for researchers. Quantitative trait locus (QTL) mapping and genome-wide association study (GWAS) have been widely applied to identify the QTLs, candidate genes and mutations affecting milk production traits in dairy cattle [5–8], and a large number of QTLs and genetic associations have been detected using such two approaches so far (http://www.animalgenome.org/cgi-bin/QTLdb/index). In recent years, short insertion and deletion (indel), as the second main form of genomic variation, has been increasingly paid more attention and has made great contribution to investigations on genetic and phenotypic diversities in human, chicken, pig and dairy cattle [9–13]. A previous study found that 2–18 base pairs (bp) indel located upstream of TAL bHLH transcription factor 1 (*TAL1*) was responsible for the T-cell acute lymphoblastic leukemia (T-ALL) [14]. In chicken, the 9–15 bp indel of premelanosome protein (*PMEL17*) gene was confirmed to be the causative mutation for the plumage color (Dominant white, Dun and Smoky) [10]. In pig, an intronic inserted retrotransposon of sperm flagellar 2 (*SPEF2*) led to the immotile short-tail sperm defect [15]. In Belgian blue cattle, a 11-bp indel in myostatin (*MSTN*) gene resulted in double-muscled phenotype [9], and an exonic 15-bp insertion in coagulation factor XI (*F11*) gene caused the factor XI deficiency in Japanese black cattle [11]. However, up to now, limited research of indel polymorphisms associated with milk production traits in dairy cattle has been reported [16].

With the rapidly emergence of next-generation sequencing (NGS), whole genome resequencing has been an important tool in the efforts to detect polymorphsims which were contributed to the complex traits or economic traits in human and domestic animals [17–20]. In our previous whole genome resequencing study, we identified over 0.9 million short indels and 3625 common differential indels with the same allelic distribution directions based on the 8 Holstein bulls with extremely high or low estimated breeding values (EBVs) of milk protein and fat percentages (high and low groups) [21]. Based on this, 11 genes were identified as the promising candidates affecting milk compositions traits in dairy cattle, including *FCGR2B*, *CENPE*, *RETSAT*, *ACSBG2*, *NFKB2*, *TBC1D1*, *NLK*, *MAP3K1*, *SLC30A2*, *ANGPT1* and *UGDH*, which contained 25 differential indels [21]. Thus, the aim of this study was to further validate whether these identified indels in the 11 genes significantly impact on milk yield and compositions traits in Chinese Holstein population.

## Results

### Indel verification and genotyping

Based on two DNA pools from 40 Holstein sires, with PCR product sequencing, 22 of 25 indels identified by whole genome resequencing [21] were confirmed as true ones (Additional file 2), among them, four indels were identified for the first time (Table 1). Subsequently, 13 indels in 8 genes were successfully genotyped and performed for association analysis. Of the 13 indels, two indels, including rs381714237 in *FCGR2B*, ss2137349053 in *CENPE* were located in the exons, whilst, the remaining 11 indels were located in the intronic regions. Chi-squared test showed that all the 13 indels were in Hardy-Weinberg equilibrium (*P* > 0.05). The genotype frequencies and allele frequencies of the 13 indels were summarized in Table 2.

**Table 1:** Detailed information of 24 indels of 11 genes identified in Chinese Holstein cattle

Indel	Gene	Location	GenBank no.	Position in UMD_3.1	Indel Sequence	Confirmed?
1 N ins	*FCGR2B*	exon7	rs381714237	chr3:7930047	G	1
3 N ins	*CENPE*	exon58	ss2137349053	chr6:23018080	AGA	1
3 N del	*CENPE*	exon68	–	chr6:23026632–23026634	TAG	3
3 N ins	*CENPE*	intron13	rs385060942	chr6:22983076	GTT	1
1 N ins	*CENPE*	intron13	ss2137349051	chr6:22983397	T	1
1 N del	*CENPE*	intron18	rs384082187	chr6:22989805	A	2
21 N ins	*CENPE*	intron24	rs377812754	chr6:22996564–22996573	ACTTAAGTATATAACCTTAAC	2
2 N del	*CENPE*	intron41	rs453960300	chr6:23018994–23018995	CC	1
1 N del	*CENPE*	intron49	rs378415122	chr6:23036105	C	1
4 N ins	*CENPE*	intron51	ss2137349056	chr6:23040582	ACAC	4
2 N del	*RETSAT*	3’UTR	rs136527375	chr11:49489416–49489417	AA	2
9 N ins	*RETSAT*	intron6	rs134985825	chr11:49485899	ATTCTGGGG	1
1 N ins	*ACSBG2*	intron7	rs377943075	chr7:19476990	G	1
2 N ins	*NFKB2*	5′ regulatory region	–	chr26:22891203	GG	3
1 N del	*TBC1D1*	intron1	rs136639319	chr6:58898979	T	1
1 N ins	*NLK*	intron1	rs137724510	chr19:20180649	T	2
2 N del	*NLK*	intron1	rs379188781	chr19:20189055–20189056	AT	1
1 N del	*NLK*	intron3	rs135129224	chr19:20264835	A	2
4 N del	*NLK*	intron3	rs134444531	chr19:20276109–20276112	AAAA	1
5 N del	*MAP3K1*	intron16	ss2137349058	chr20:22365627–22365631	CATTT	1
6 N del	*SLC30A2*	intron2	ss2137349049	chr2:127640012–127640017	TTTTTG	2
2 N ins	*ANGPT1*	intron1	ss2137349057	chr14:59305051	AT	2
1 N ins	*UGDH*	intron7	rs383327605	chr6:60236955	T	2
1 N ins	*UGDH*	intron2	ss2019489562	chr6:60252782	G	1

Note: ^1^ indels were genotyped successfully; ^2^ indels were failed to genotype using MALDI-TOF MS; ^3^ indels were not polymorphic in current population; ^4^ primers of indel were failed to design

**Table 2:** The genotypic and allelic frequencies of 13 indels of 8 genes

Locus	Gene	Genotype	Genotype frequencies	Allele	Allele frequencies
rs381714237	*FCGR2B*	del/del	0.115	del	0.369
		del/ins	0.508	ins	0.631
		ins/ins	0.377		
ss2137349053	*CENPE*	del/del	0.208	del	0.462
		del/ins	0.507	ins	0.538
		ins/ins	0.284		
rs385060942	*CENPE*	del/del	0.206	del	0.462
		del/ins	0.512	ins	0.538
		ins/ins	0.282		
ss2137349051	*CENPE*	del/del	0.277	del	0.534
		del/ins	0.513	ins	0.466
		ins/ins	0.210		
rs453960300	*CENPE*	ins/ins	0.212	ins	0.467
		ins/del	0.511	del	0.533
		del/del	0.277		
rs378415122	*CENPE*	ins/ins	0.210	ins	0.467
		ins/del	0.514	del	0.533
		del/del	0.276		
rs134985825	*RETSAT*	del/del	0.276	del	0.524
		del/ins	0.497	ins	0.476
		ins/ins	0.227		
rs377943075	*ACSBG2*	del/del	0.082	del	0.276
		del/ins	0.388	ins	0.724
		ins/ins	0.530		
rs136639319	*TBC1D1*	ins/ins	0.025	ins	0.186
		ins/del	0.321	del	0.814
		del/del	0.654		
rs379188781	*NLK*	ins/ins	0.374	ins	0.608
		ins/del	0.468	del	0.392
		del/del	0.158		
rs134444531	*NLK*	ins/ins	0.265	ins	0.532
		ins/del	0.532	del	0.468
		del/del	0.202		
ss2137349058	*MAP3K1*	ins/ins	0.207	ins	0.466
		ins/del	0.518	del	0.534
		del/del	0.275		
ss2019489562	*UGDH*	del/del	0.220	del	0.466
		del/ins	0.491	ins	0.534
		ins/ins	0.289		

### Associations between indels and five milk production traits

The results of associations between the 13 indels and five milk production traits were shown in Table 3. It was observed that all these indels were significantly associated with at least one of the milk traits (*P* < 0.0001 to *P =* 0.0312) as described below.

**Table 3:** Association results of the thirteen indels in eight genes on the five milk production traits (least squares mean ± SE)

Locus	Gene	Genotype (No.)	MY	FY	FP	PY	PP
rs381714237	*FCGR2B*	del/del(86)	10,891 ± 99.80	385.05 ± 4.21	3.59 ± 0.040	319.32 ± 3.10^ab^	2.95 ± 0.014a
		ins/del(380)	10,702 ± 64.80	383.94 ± 2.89	3.66 ± 0.027	315.86 ± 2.11^Aa^	2.98 ± 0.009^ab^
		ins/ins(282)	10,834 ± 68.20	385.61 ± 3.03	3.64 ± 0.028	321.83 ± 2.20^Bb^	2.99 ± 0.009^b^
		*P value*	0.0297*	0.7926	0.1738	0.0041**	0.0198*
ss2137349053	*CENPE*	del/del(153)	10,403 ± 55.66^A^	374.36 ± 2.82^Aa^	3.61 ± 0.034	308.40 ± 2.65^A^	2.97 ± 0.024
		del/ins(373)	10,661 ± 47.88^B^	378.61 ± 2.43^Aa^	3.57 ± 0.027	315.70 ± 2.14^Ba^	2.97 ± 0.017
		ins/ins(209)	10,852 ± 51.03^C^	385.99 ± 2.59^B^	3.58 ± 0.031	321.05 ± 2.37^Bb^	2.97 ± 0.020
		*P value*	< 0.0001**	< 0.0001**	0.3733	< 0.0001**	0.9882
rs385060942	*CENPE*	del/del(152)	10,494 ± 84.00^Aa^	376.49 ± 3.60^Aa^	3.60 ± 0.034	310.45 ± 2.62^B^	2.96 ± 0.012
		del/ins(377)	10,717 ± 65.77^Aa^	381.44 ± 2.93^ab^	3.57 ± 0.027	318.18 ± 2.13^Aa^	2.97 ± 0.009
		ins/ins(208)	10,871 ± 74.83^B^	387.82 ± 3.26^Bb^	3.59 ± 0.031	322.24 ± 2.38^Aa^	2.97 ± 0.010
		*P value*	< 0.0001**	0.0045**	0.6063	< 0.0001**	0.6166
ss2137349051	*CENPE*	ins/ins(206)	10,817 ± 75.26^A^	386.81 ± 3.28^A^	3.60 ± 0.031	321.11 ± 2.39^A^	2.97 ± 0.010
		del/ins(382)	10,586 ± 65.52^Ba^	378.41 ± 2.92^Ba^	3.59 ± 0.027	314.42 ± 2.12^Ba^	2.98 ± 0.009
		del/del(156)	10,397 ± 83.94^Bb^	374.49 ± 3.61^Ba^	3.61 ± 0.034	308.61 ± 2.63^Bb^	2.97 ± 0.012
		*P value*	< 0.0001**	0.0010**	0.7289	< 0.0001**	0.9571
rs453960300	*CENPE*	ins/ins(158)	10,380 ± 83.33^Aa^	375.15 ± 3.58	3.63 ± 0.034	307.63 ± 2.61^Aa^	2.98 ± 0.012
		ins/del(381)	10,586 ± 66.10^b^	380 ± 2.95	3.61 ± 0.027	313.34 ± 2.15^b^	2.98 ± 0.009
		del/del(207)	10,764 ± 74.83^Bc^	385.96 ± 3.26	3.61 ± 0.031	318.18 ± 2.38^Bc^	2.97 ± 0.010
		*P value*	< 0.0001**	0.0073**	0.7589	0.0002**	0.9461
rs378415122	*CENPE*	ins/ins(158)	10,373 ± 83.79^Aa^	373.03 ± 3.60^Aa^	3.62 ± 0.034	306.81 ± 2.62^Aa^	2.97 ± 0.012
		ins/del(386)	10,547 ± 65.79^Aa^	377.75 ± 2.93^a^	3.60 ± 0.027	312.15 ± 2.13^Aa^	2.97 ± 0.009
		del/del(207)	10,785 ± 74.23^B^	386.21 ± 3.23^Bb^	3.60 ± 0.030	319.19 ± 2.36^B^	2.97 ± 0.010
		*P value*	< 0.0001**	0.0004**	0.8913	< 0.0001**	0.9535
rs134985825	*RETSAT*	del/del(205)	10,774 ± 75.77^Aa^	383.94 ± 3.30^ab^	3.58 ± 0.031	318.23 ± 2.40^ab^	2.96 ± 0.010^a^
		del/ins(370)	10,563 ± 66.51^B^	378.10 ± 2.95^Aa^	3.60 ± 0.027	314.44 ± 2.15^Aa^	2.98 ± 0.009^ab^
		ins/ins(169)	10,823 ± 79.45^Aa^	388.92 ± 3.43^Bb^	3.61 ± 0.032	323.7 ± 2.50^Bb^	2.99 ± 0.011^b^
		*P value*	0.0003**	0.0009**	0.6500	0.0001**	0.0237*
rs377943075	*ACSBG2*	del/del(62)	10,746 ± 111.22	378.40 ± 4.68^Aa^	3.56 ± 0.045	318.61 ± 3.42	3.00 ± 0.016^ab^
		del/ins(294)	10,776 ± 69.04	387.18 ± 3.03^ab^	3.61 ± 0.028	319.22 ± 2.19	2.99 ± 0.009^Ab^
		ins/ins(401)	10,884 ± 63.81	391.00 ± 2.84^Bb^	3.62 ± 0.026	319.31 ± 2.05	2.97 ± 0.008^Ba^
		*P value*	0.1334	0.0088**	0.4393	0.9749	0.0052**
rs136639319	*TBC1D1*	ins/ins(19)	10,752 ± 182.68	391.92 ± 7.47	3.65 ± 0.073^ab^	320.14 ± 5.45	2.98 ± 0.026^ab^
		del/ins(242)	10,564 ± 72.44	383.75 ± 3.18	3.65 ± 0.030^a^	314.38 ± 2.32	2.99 ± 0.010^a^
		del/del(493)	10,659 ± 64.49	380.06 ± 2.88	3.58 ± 0.027^b^	314.42 ± 2.1	2.96 ± 0.009^b^
		*P value*	0.2493	0.1198	0.0224*	0.5384	0.0209*
rs379188781	*NLK*	ins/ins(277)	10,676 ± 70.45	377.09 ± 3.11	3.56 ± 0.029	318.23 ± 2.26	2.99 ± 0.009^Aa^
		del/ins(347)	10,627 ± 67.41	381.09 ± 3.00	3.60 ± 0.028	314.48 ± 2.18	2.97 ± 0.009^b^
		del/del(117)	10,749 ± 90.28	384.24 ± 3.83	3.57 ± 0.037	315.92 ± 2.79	2.95 ± 0.013^Bb^
		*P value*	0.3389	0.1020	0.2615	0.1279	0.0047**
rs134444531	*NLK*	ins/ins(197)	10,748 ± 78.14	384.18 ± 3.40	3.58 ± 0.032	322.04 ± 2.47^Aa^	2.99 ± 0.011^a^
		del/ins(395)	10,723 ± 65.31	386.23 ± 2.91	3.61 ± 0.027	317.93 ± 2.12^a^	2.97 ± 0.009^ab^
		del/del(150)	10,563 ± 85.88	381.58 ± 3.68	3.63 ± 0.035	312.32 ± 2.68^Bb^	2.96 ± 0.012^b^
		*P value*	0.0733	0.3026	0.4078	0.0012**	0.0257*
ss2137349058	*MAP3K1*	ins/ins(152)	10,482 ± 82.27^Aa^	372.63 ± 3.54^Aa^	3.58 ± 0.034	310.01 ± 2.58^Aa^	2.97 ± 0.011
		del/ins(380)	10,613 ± 66.30^Aa^	376.47 ± 2.94^Aa^	3.56 ± 0.027	314.12 ± 2.14^Aa^	2.97 ± 0.009
		del/del(202)	10,909 ± 77.27^Bb^	390.47 ± 3.35^Bb^	3.59 ± 0.032	323.13 ± 2.44^Bb^	2.97 ± 0.011
		*P value*	< 0.0001**	< 0.0001**	0.4863	< 0.0001**	0.8495
ss2019489562	*UGDH*	del/del(405)	10,860 ± 64.35^a^	387.55 ± 2.89	3.64 ± 0.027	318.31 ± 2.11	2.99 ± 0.009
		del/ins(4)	9902 ± 384.75^b^	376.58 ± 15.49	3.81 ± 0.153	299.74 ± 11.30	3.02 ± 0.056
		ins/ins(342)	10,798 ± 66.31^ab^	388.28 ± 2.95	3.64 ± 0.027	315.05 ± 2.13	2.97 ± 0.009
		*P value*	0.0312*	0.7267	0.5077	0.0532	0.0567

Note:*significant association at the significance level of 0.05; **significant association at the significance level of 0.01

The different superscripts (^A,B^ within the same column with different superscripts indicate *P* < 0.01; ^a,b^ indicate *P* < 0.05) adjusted after correction for multiple testing indicate significant differences among the genotypes

*MY* milk yield, *FY* fat yield, *FP* fat percentage, *PY* protein yield, *PP* protein percentage

### Exonic indels

The exonic indel rs381714237 in *FCGR2B* was associated with milk yield (*P* = 0.0297), protein yield (*P* = 0.0041) and protein percentage (*P* = 0.0198). The other exonic indel ss2137349053 in *CENPE,* was strongly associated with milk yield (*P* < 0.0001), fat yield (*P* < 0.0001) and protein yield (*P* < 0.0001).

### Intronic indels

**Fig. 1:**
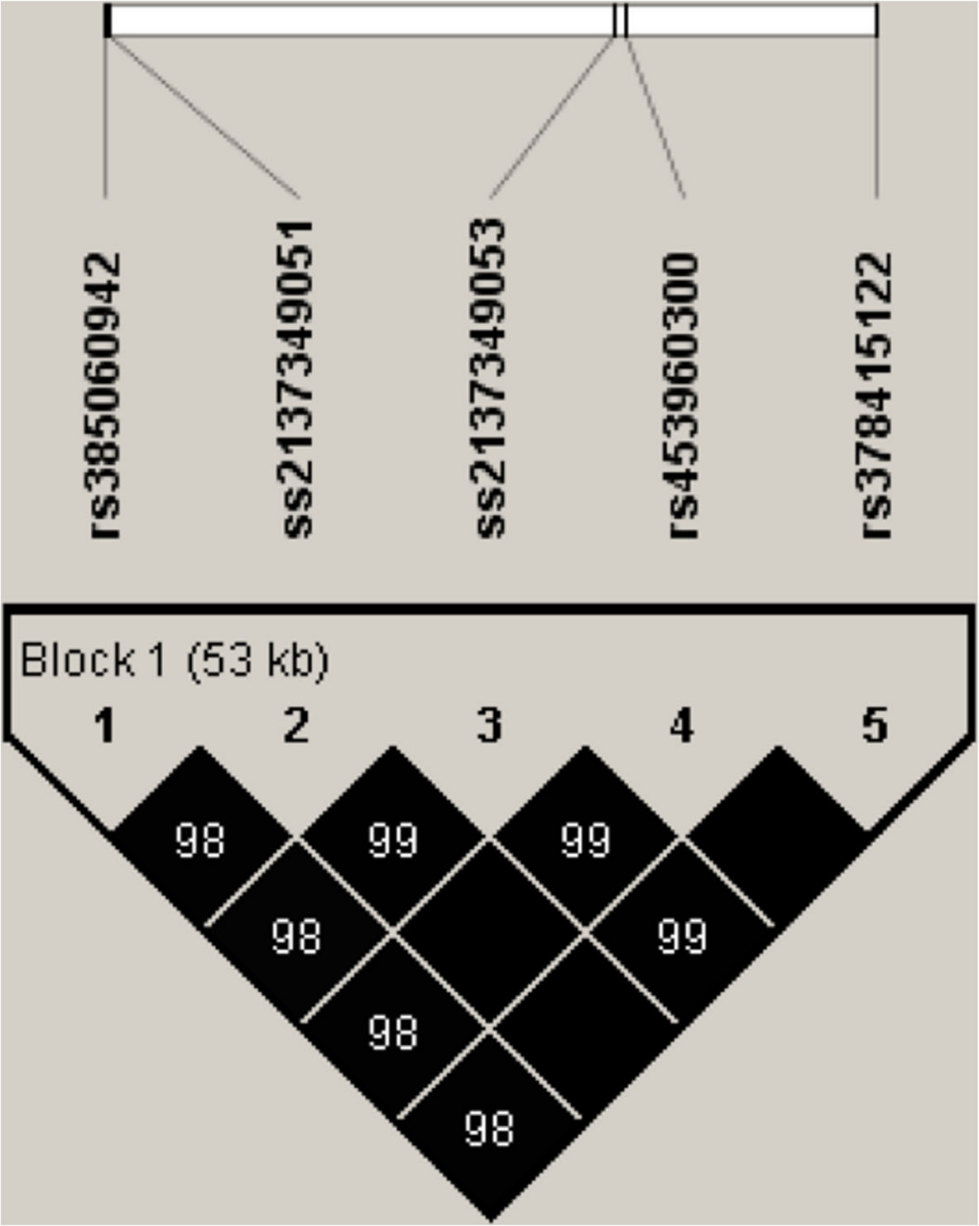
Linkage disequilibrium estimated of the five indels in *CENPE* gene. The values in boxes are pair-wise indel correlations (r^2^)

The four intronic indels (rs385060942, ss2137349051, rs453960300 and rs378415122) in *CENPE* were significantly associated with milk yield (*P* < 0.0001), fat yield (*P* = 0.0004 to 0.0073) and protein yield (*P* < 0.0001 to 0.0002). Additionally, the five indels (four intronic indels and one exonic indel above) of *CENPE* gene were found to be highly linked (r^2^ > 0.98), and one haplotype block was inferred as presented in Fig. 1. Haplotype-based association analysis showed that the haplotype combination was evidently associated with milk yield, fat yield and protein yield as well (*P* < 0.0001 to *P* = 0.0076) (Table 4).

**Table 4:** Haplotype analysis of *CENPE* gene (least squares mean ± SE)

Haplotype (No.)	MY	FY	FP	PY	PP
H1H1 (198)	10,874 ± 77.45^Aa^	389.53 ± 3.38^Aa^	3.60 ± 0.031	321.43 ± 2.46^Aa^	2.97 ± 0.011
HIH2 (356)	10,734 ± 68.09^Aa^	382.37 ± 3.02^b^	3.58 ± 0.028	316.68 ± 2.20^Ab^	2.96 ± 0.009
H2H2 (140)	10,498 ± 88.34^Bb^	378.32 ± 3.78^Bb^	3.62 ± 0.036	309.40 ± 2.75^B^	2.95 ± 0.012
*P value*	0.0003**	0.0076**	0.4045	< 0.0001**	0.7803

Note:*significant association at the significance level of 0.05; **significant association at the significance level of 0.01

The different superscripts (^A,B^ within the same column with different superscripts indicate *P* < 0.01; ^a,b^ indicate P < 0.05) adjusted after correction for multiple testing indicate significant differences among the genotypes

*MY* milk yield, *FY* fat yield, *FP* fat percentage, *PY* protein yield, *PP* protein percentage

Indel rs134985825 in the intron 6 of *RETSAT* showed remarkable effects on milk yield, protein yield, fat yield and protein percentage (*P* = 0.0001 to 0.0237). For *ACSBG2*, indel rs377943075 in the intron 7 was significantly associated with fat yield (*P* = 0.0088) and protein percentage (*P* = 0.0052). Variant rs136639319 in the intron 3 of *TBC1D1*was significantly associated with fat percentage (*P* = 0.0224) and protein percentage (*P* = 0.0209).

For the indel rs379188781 in the intron 1 of *NLK*, it was found to be associated with protein percentage (*P* = 0.0047), the other indel rs134444531 in the intron 3 was associated with protein yield (*P* = 0.0012) and protein percentage (*P* = 0.0257). While, no LD was observed between such two indels (r^2^ = 0.14).

For *MAP3K1*, indel ss2137349058 in the intron 16 was markedly associated with milk yield, fat yield and protein yield (*P* < 0.0001).

For the intronic indel of *UGDH* gene, the indel ss2019489562 located in intron 2 was significantly associated with milk yield (*P* = 0.0312).

Additionally, the significant additive, dominant and allele substitution effects of the 13 indels on the five milk traits were observed as well (Table 5).

**Table 5:** Genetic effects of thirteen indels in eight genes on five milk production traits

Locus	Gene	Gene effects	MY	FY	FP	PY	PP
rs381714237	*FCGR2B*	Additive effect(a)	66.13*	0.83	−0.0072	2.99**	0.0051
		Dominant effect(d)	123.27	0.27	− 0.0618	0.47	−0.0348**
		Substitution effect(α)	30.92	0.76	0.0105	2.85*	0.0150*
ss2137349053	*CENPE*	Additive effect(a)	224.66**	5.81**	−0.0174	6.32**	− 0.0002
		Dominant effect(d)	33.43	−1.57	−0.0246	0.98	0.0028
		Substitution effect(α)	227.29**	5.69**	−0.0193	6.4**	0.0001
rs385060942	*CENPE*	Additive effect(a)	−188.63**	−5.66**	0.007	−5.89**	−0.0042
		Dominant effect(d)	34.37	−0.71	−0.0218	1.84	0.0065
		Substitution effect(α)	−191.51**	−5.60**	0.0089	−6.05**	−0.0048
ss2137349051	*CENPE*	Additive effect(a)	−209.61**	− 6.16**	0.0093	− 6.25**	−0.0009
		Dominant effect(d)	−20.82	− 2.24	−0.014	−0.44	0.0022
		Substitution effect(α)	−208.06**	−6.00**	0.0103	−6.22**	−0.0011
rs453960300	*CENPE*	Additive effect(a)	− 192.46**	−5.40**	0.0105	− 5.27**	0.0015
		Dominant effect(d)	13.90	−0.55	−0.0106	0.43	0.0017
		Substitution effect(α)	−193.46**	−5.36**	0.0113	−5.31**	0.0014
rs378415122	*CENPE*	Additive effect(a)	−205.95**	−6.59**	0.0069	−6.19**	−0.0011
		Dominant effect(d)	−32.03	−1.87	−0.0068	− 0.85	0.0022
		Substitution effect(α)	−203.68**	−6.46**	0.0073	−6.13**	−0.0012
rs134985825	*RETSAT*	Additive effect(a)	24.57	2.49	0.0156	2.74*	0.0163**
		Dominant effect(d)	− 235.31**	−8.32**	0.0001	−6.52**	0.0036
		Substitution effect(α)	29.53	2.67	0.0156	2.88*	0.0162**
rs377943075	*ACSBG2*	Additive effect(a)	−69.28	−6.30**	−0.0272	−0.35	0.0154*
		Dominant effect(d)	−39.32	2.48	0.0178	0.26	0.0108
		Substitution effect(α)	−56.34	−7.12*	−0.033	−0.44	0.0118
rs136639319	*TBC1D1*	Additive effect(a)	47.54	−1.85	−0.0342**	0.02	−0.0127**
		Dominant effect(d)	140.15	10.02	0.0346	5.74	0.0103
		Substitution effect(α)	98.36	1.79	−0.0216	2.11	−0.0089
rs379188781	*NLK*	Additive effect(a)	−36.45	−3.58	−0.0057	1.16	0.0194**
		Dominant effect(d)	−85.74	0.43	0.0343	−2.59	−0.0039
		Substitution effect(α)	−55.71	−3.48	0.002	0.58	0.0185*
rs134444531	*NLK*	Additive effect(a)	92.55	1.30	−0.0226	4.86**	0.0169*
		Dominant effect(d)	67.33	3.36	0.0084	0.75	−0.0055
		Substitution effect(α)	97.88	1.57	−0.0219	4.92**	0.0164*
ss2137349058	*MAP3K1*	Additive effect(a)	−213.49**	−8.92**	−0.0046	−6.56**	0.0013
		Dominant effect(d)	−82.74	−5.08*	−0.0264	−2.45	0.0043
		Substitution effect(α)	−208.37**	−8.60**	−0.0029	−6.41**	0.0011
ss2019489562	*UGDH*	Additive effect(a)	31.00	−0.36	− 0.0032	1.63	0.0097*
		Dominant effect(d)	−925.95*	−11.33	0.1715	−16.94	0.0462
		Substitution effect(α)	−42.62	−1.27	0.0104	0.28	0.0134*

Note: (a), (d), (α) means Additive, Dominant and Substitution effect, respectively

* means the additive, dominant or allele substitution effect of the locus indicated differ at *P* < 0.05 and ** means the additive, dominant or allele substitution effect of the locus indicated differ at *P* < 0.01

*MY* milk yield, *FY* fat yield, *FP* fat percentage, *PY* protein yield, *PP* protein percentage

### Prediction the mRNA and protein structures

**Fig. 2:**
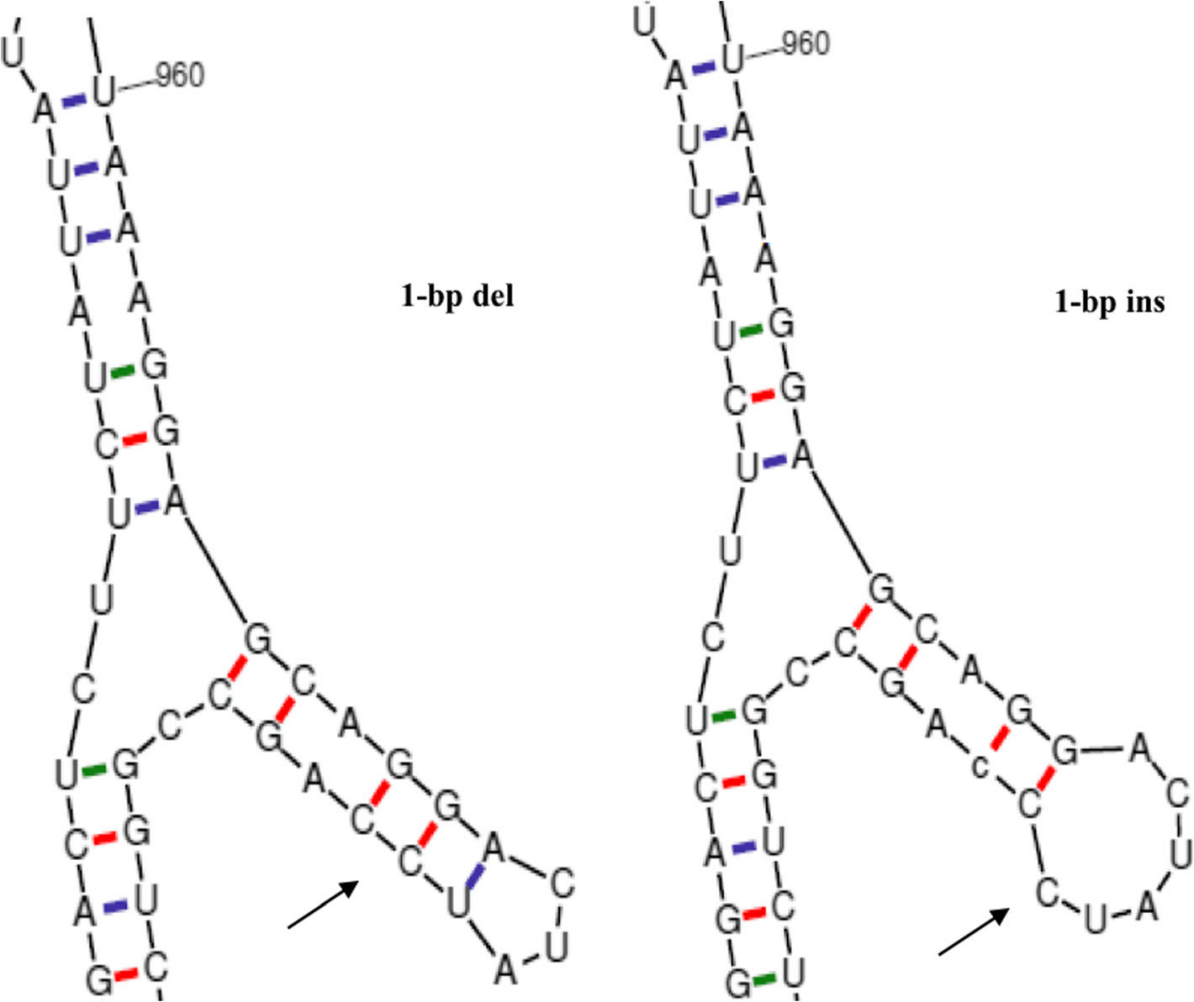
The predicted mRNA secondary structures corresponding to the exonic indel of *FCGR2B* gene

Using a statistical folding algorithm, the alteration of the most stable mRNA secondary structures caused by the two exonic indels for *FCGR2B* and *CENPE* were observed for both the ins/ins and del/del genotypes. As illustrated in Fig. 2, obvious structural differences spanning the position 971–980 between the ins/ins and del/del genotypes of the indel rs381714237 in *FCGR2B* gene were observed. The free energy (∆G) of the ins allele was predicted to be higher (∆G = − 468.70 kcal/mol) than the del allele (∆G = − 470.30 kcal/mol). Correspondingly, the ins allele was deduced to form one larger single loop structure, which potentially decreasing the stability of mRNA (∆∆G = + 1.6 kcal/mol). It is worth mentioning that previous studies have evidenced that the ∆∆G ranged from − 3.9 kcal/mol to + 4.0 kcal/mol could affect the mRNA stability [22–28]. In addition, indel rs381714237 of *FCGR2B* was predicted to decrease the number of amino acid by 38, which might change protein structure and function. As a result, differences of the protein secondary structures were predicted between the FCGR2B proteins corresponding to alleles del and ins with regard to alpha helix (21.64% vs. 16.45%), extended strand (23.10% vs. 24.67%), beta turn (7.02% vs. 7.89%) and random coil (48.25% vs. 50.99%) using the SOPMA program.

For the non-frameshiting indel, subtle change of mRNA secondary structures between the two homozygous genotypes of indel ss2137349053 in *CENPE* was occurred (data not shown). The free energy was altered from − 1816.10 kcal/mol for the del allele to − 1818.90 kcal/mol for the ins allele. While, slight difference was predicted for the CENPE protein in accordance between the del/del and ins/ins genotypes, alpha helix (72.57% vs. 72.69%), and random coil (13.94% vs. 13.82%). There was no change of extended strand and beta turn for CENPE protein.

## Discussion

In the present work, we confirmed that 13 indels belonging to 8 candidate genes (*FCGR2B*, *CENPE*, *RETSAT*, *ACSBG2*, *TBC1D1*, *NLK*, *MAP3K1* and *UGDH*) for milk compositions identified by our previous whole genome resequencing study [21] showed significant genetic effects on at least one of milk traits in dairy cattle. As far as our knowledge, this is the first report to connect these genes to milk production traits of dairy cattle.

Among the total 25 differential indels with the same allelic distribution directions between the bulls in high and low groups identified by our previous whole genome resequencing study [21], indel rs383700527 (3 N ins) located upstream of *ACSBG2* gene was found to contribute to milk fat in a cis-regulatory manner (unpublished data). Thus, we investigated another 24 indels in the present study. Among them, one intronic indel (4 N ins in *CENPE*) was failed to be verified by Sanger sequencing due to the special characteristic of the flanking sequence with lower GC% and repetitive DNA sequences. Two indels (3 N del in *CENPE* and 2 N ins in *NFKB2*) didn’t show polymorphic in this study. Eight indels (1 N del and 21 N ins in *CENPE*, 2N del in *RETSAT*, 1 N ins and 1 N del in *NLK*, 6 N del in *SLC30A2,* 2 N ins in *ANGPT1* and 1N ins in *UGDH*) were failed to be genotyped by using MALDI-TOF MS. The possible reason may be that MALDI-TOF MS for multiplex genotyping was relied on multiplex-PCR primers and extended primers to genotype multiple loci [29], simultaneously, the primer design was depended on sequence composition, molecular weight, annealing temperature and reaction efficiencies of each locus [29]. Hence, a total of 13 polymorphic indels were successfully genotyped and performed for association analysis.

### Significant associations between candidate genes and milk production traits

#### Six indels in *FCGR2B* and *CENPE*

For indel rs381714237 in *FCGR2B*, we demonstrated that ins/ins genotype had higher protein percentage. As a regulator, FCGR2B was contributed to immune response [30]. Additionally, bovine mammary gland is a product of the innate immune system and active during lactation. Thus, these evidences indicated that *FCGR2B* might affect milk protein percentage through impacting the cows on immune response during lactation.

For the five indels in *CENPE*, the association results revealed that ins/ins genotypes were dominant compared with del/del genotypes for milk yield, fat yield and protein yield. Previous report has found that CENPE acted as a monitor protein and was necessary for cell cycle [31]. Thus, it appeared that the *CENPE* might affect these traits through modulating bovine mammary gland development.

### Seven indels located in six genes

Our association analysis confirmed that the ins/ins genotype of the indel rs134985825 in *RETSAT* gene increased milk yield, fat yield, and protein yield. *RETSAT* was considered as a regulator for liver metabolism, and was critical for lipid accumulation and adipogenesis promotion [32]. Previous research has investigated that the polymorphisms of *RETSAT* gene were associated with premium cut yields and backfat thickness in pig [33]. Taken together, we speculated that *RETSAT* might affect milk traits through influencing the lipid metabolism.

Herein, we found that individuals with ins/ins genotype of indel rs377943075 in *ACSBG2* showed higher fat yield than those with del/del genotype. The *ACSBG2* gene encodes the protein that belongs to a member of the acyl-CoA synthetase family and participated in PPAR signaling pathway and involved in lipid metabolism and lipid droplet formation [34, 35]. Previous researchers have found that polymorphisms of *ACSBG2* showed positive effects on yolk development and abdominal fat weight [36].

In current study, our results also showed a significant relationship between the indel rs136639319 in *TBC1D1* and fat percentage as well as protein percentage. It was worth mentioning that TBC1D1, as a member of Rab GTPase-activating proteins (GAPs), was involved in translocation of GLUT4 to the plasma membrane. Polymorphisms in *TBC1D1* have been observed to show significant effects on severe obesity or carcass in human [37] and chicken [38], respectively, suggesting exhibiting functions related to lipid and energy homeostasis as reported by Hargett et al. [39, 40].

Two intronic indel (rs379188781 and rs134444531) in *NLK* showed strong associations with protein yield and protein percentage. Interestingly, Cole et al. reported that one single nucleotide polymorphism (SNP) (ARS-BFGL-NGS-106227) significantly associated with protein percentage (*P* = 5.59 × 10^− 8^) was merely 90 kb away from the *NLK* gene [41]. Furthermore, *NLK*, as a member of MAPK subfamily, had an essential role in mediating the mTORC1 signaling pathway which was involved in milk protein synthesis [42, 43]. Together, these data suggested that significant variation of protein yield and protein percentage might be regulated by *NLK*.

As for *MAP3K1*, individuals with del/del genotype of indel polymorphism ss2137349058 had higher milk yield, fat yield and protein yield. *MAP3K1* was known to be involved in the MAPK signaling pathway, and was considered to be a metabolic stimuli inducing cell proliferation [44, 45]. Meanwhile, it functioned as a candidate gene for type 2 diabetes (T2D) by interacting with insulin signaling pathway [46]. Thus, we concluded that *MAP3K1* might regulate milk composition traits by modulating bovine mammary gland development.

The intronic indel ss2019489562 in *UGDH* showed significant effect on milk yield. *UGDH* encodes the protein that was implicated with biosynthesis of glycosaminoglycans, hyaluronan, chondroitin sulfate, and heparan sulfate. Previously, Xu et al. have demonstrated that two exonic SNPs in *UGDH* showed significant associations with milk production traits in Chinese Holstein population [47]. In particular, *UGDH* was close to the peak location of two reported QTLs for fat yield, fat percentage and protein yield [48–51]. Further, two previously reported significant SNPs for fat yield, protein yield, fat percentage and protein percentage [41] were near to the *UGDH* gene. Moreover, expression pattern in InnateDB showed that *UGDH* have the highest expression in liver which plays an indispensable role in metabolism of carbohydrates, fats and proteins in dairy cattle. Hence, these data demonstrated that *UGDH* gene might be a vital regulator for milk traits by affecting liver metabolism.

## Conclusion

In the present study, we performed association analysis for the 13 short indels within 8 candidate genes for milk compositions identified by our previous whole genome resequencing study, including *FCGR2B*, *CENPE*, *RETSAT*, *ACSBG2*, *TBC1D1, NLK*, *MAP3K1* and *UGDH*. As a result, the 13 indels were shown to have significant genetic effects on at least one of milk yield and composition traits. These results not only validated the candidate genes and indels from the previous whole genome resequencing work, but also provided novel molecular information for genetic improvement program of dairy cattle.

## Methods

### Ethics statement

All the procedures for sample collections and phenotypic observations of experimental individuals were carried out along with regular quarantine inspection of the farms and in strict accordance with the protocol reviewed and approved by the Institutional Animal Care and Use Committee (IACUC) at China Agricultural University, and the permit number is DK996.

### Animals

The animals used for association analysis included a total of 769 Chinese Holstein cows those were daugters of 40 sire families. These daughters were collected from 22 herds of Beijing Sanyuanlvhe Dairy Farming Center, a leading dairy company in China. Phenotypic data of the five milk production traits including 305-day milk yield (MY), fat yield (FY), protein yield (PY), fat percentage (FP) and protein percentage (PP) those were calculated based on at least 6 test-day records in each lactation using a multiple trait random regression test-day model by the Dairy Data Center of China, Dairy Association of China (http://www.holstein.org.cn/).

Genomic DNA was isolated from whole blood of cows and frozen semen of sires as previously described by Yang et al. [16].

### Indels selection, PCR amplification, sequencing and genotyping

Of the 25 short indels that identified by our previous whole-genome resequencing study, 24 indels were investigated the associations with the five milk production traits except for a three-nucleotide insertion (3 N ins) in *ACSBG2* gene.

A total of 23 pairs of PCR primers were designed with Primer Premier 5.0 and Oligo 7.0 softwares based on the genomic sequences of the 11 candidate genes in Bos_taurus_UMD3.1 assembly (Additional file 1). To identify the twenty-four potential indel polymorphisms, two DNA pools for the above 40 sires were constructed with equal concentration of 50 ng/μl of each bull (20 individuals/pool). PCR products basd on the pooled DNA were purified with an EasyPure PCR Purification Kit (TransGen Biotech, Beijing, China) and then bi-directionally sequenced using ABI3730xl DNA Analyzer (Applied Biosystems, Foster City, CA, USA).

To further confirm the position and sequence of the insertions and deletions, the purified PCR products were cloned into the pClone007 vector with a pClone 007 Vector Kit (TsingKe Biological Technology, Beijing, China). Positive clones including target indels were sequenced to search potential indels. The BLAST software (https://blast.ncbi.nlm.nih.gov/Blast.cgi) and Chromas 2.0 (Technelysium, Australia) were applied for sequence alignment to the reference sequence of the corresponding gene referring to Bos_taurus_UMD_3.1 assembly. Finally, genotyping for the identified indels in 769 chinese cows was performed by using the Sequenom MassArray matrix-assisted laser desorption ionization time-of-flight mass spectrometry (MALDI-TOF MS).

### Bioinformatics analysis

To further explore the potential impact of the exonic indels in *FCGR2B* and *CENPE* on the mRNA secondary structures as well as the second structures of corresponding proteins, the online RNA FOLDING FORM (version 2.3) software [52] and SPOMA program (http://npsa-pbil.ibcp.fr/) [53] were used, respectively.

### Association analysis

Allele frequencies and genotype frequencies between the insertion and deletion genotypes, as well as the Hardy-Weinberg equilibrium were determined through a chi-square test. Associations between the 13 investigated indels and the five milk production traits were carried out by applying the mixed procedure in SAS 9.2 [54] based on the following linear mixed regression model:

$$ \mathrm{Y}=\kern0.5em \upmu \kern0.5em +\kern0.5em \mathrm{hys}\kern0.5em +\kern0.5em \mathrm{b}\kern0.5em +\kern0.5em \mathrm{M}\kern0.5em +\kern0.5em \mathrm{G}\kern0.5em +\kern0.5em \mathrm{a}\kern0.5em +\kern0.5em \mathrm{e} $$

Where Y is the phenotypic record for the analyzed trait of the cows, μ is the overall mean of the phenotypic record for each trait, hys is a fixed effect of herd, year and season, b is linear regression coefficient on calving month (M), M is effect of calving month, G is a fixed effect of indel genotype or haplotype, a is a random polygenic effect account for all known pedigree relationships, and e is a random residual.

Also, we estimated the additive (a), dominance (d) and allele substitution (α) effects using the equation of Falconer & Mackay [55]:$$ \mathrm{a}=\raisebox{1ex}{$\left(\mathrm{AA}\hbox{-} \mathrm{BB}\right)$}\!\left/ \!\raisebox{-1ex}{$2$}\right.,\mathrm{d}=\mathrm{AB}\hbox{-} \raisebox{1ex}{$\mathrm{AA}+\mathrm{BB}$}\!\left/ \!\raisebox{-1ex}{$2$}\right.\kern0.5em \mathrm{and}\kern0.5em \upalpha =\mathrm{a}+\mathrm{d}\left(\mathrm{q}\hbox{-} \mathrm{p}\right) $$ where AA, BB and AB were the least square means of the phenotypic values for corresponding genotypes, and p and q indicates the allele frequencies of the corresponding alleles. Multiple t-tests with Bonferroni correction were used to compare the effects of the genotypes on each indel.

The linkage disequilibrium (LD) extent among the genotyped indels (five indels in *CENPE* gene and two indels in *NLK* gene) and haplotype blocks was estimated using Haploview 4.2 (Broad Institute of MIT and Harvard, Cambridge, MA, USA).

## Additional files

Additional file 1: **Table S1.** Primers used for pooled DNA sequencing for the 24 indels. (XLSX 42 kb)

Additional file 2: Results of sanger and clone sequencing of the thirteen indels. (DOCX 332 kb)

## Data Availability

All relevant data are available within the article and its additional files.

## Abbreviations

*ACSBG2*: Acyl-CoA synthetase bubblegum family member 2
bp: Base pair
*CENPE*: Centromere protein E
EBV: Estimated breeding value
*F11*: Coagulation factor XI
*FCGR2B*: Fc fragment of IgG receptor IIb
FP: Fat percentage
FY: Fat yield
GWAS: Genome-wide association study
indel: Insertion and deletion
MALDI-TOFMS: Matrix-assisted laser desorption/ionization time of flight mass spectrometry
*MAP3K1*: Mitogen-activated protein kinase kinase kinase 1
*MSTN*: Myostatin
MY: Milk yield
NGS: Next-generation sequencing
*NLK*: Nemo like kinase
*PMEL17*: Premelanosome protein
PP: Protein percentage
PY: Protein yield
QTL: Quantitative trait locus
*RETSAT*: Retinol saturase
SNP: Single nucleotide polymorphism
*SPEF2*: Sperm flagellar 2
T2D: Type 2 diabetes
*TAL1*: TAL bHLH transcription factor 1
T-ALL: T-cell acute lymphoblastic leukemia
*TBC1D1*: TBC1 domain family member 1
*UGDH*: UDP-glucose 6-dehy- drogenase
