# Supplementary material for: Determination of genetic associations between indels in 11 candidate genes and milk composition traits in Chinese Holstein population
Source: BMC Genet. 2019 May 28;20:48. doi: 10.1186/s12863-019-0751-y (PMC6537361; doi:10.1186/s12863-019-0751-y)
Supplement: Supplementary file 2 — Results of sanger and clone sequencing of the thirteen indels. (DOCX 332 kb) [file 12863_2019_751_MOESM2_ESM.docx]

**Figure. S1. Results of sanger and clone sequencing of the thirteen indels.**

The RS indicates the reference sequence; Ins and Del represent the sequence with insertion/deletion identified in this study, respectively.

rs381714237 ss2137349053


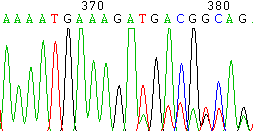

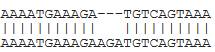


RS

Ins


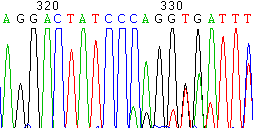

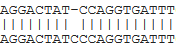


Ins

RS

rs385060942 ss2137349051


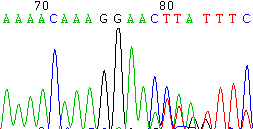

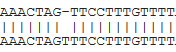


Ins

RS


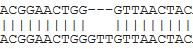

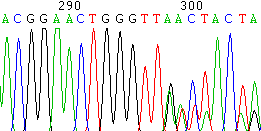


Ins

RS

rs453960300 rs378415122


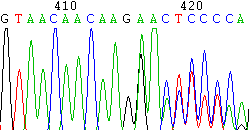


Del

RS


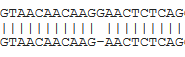

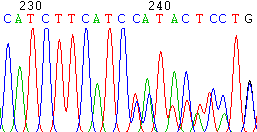

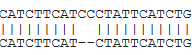


RS

Del

rs134985825 rs377943075


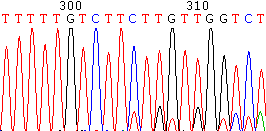


RS

Ins


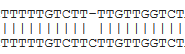

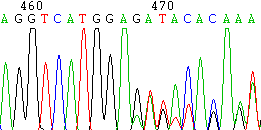

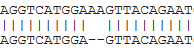


Del

RS

rs136639319 rs379188781


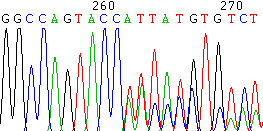


RS

Del


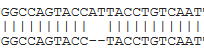

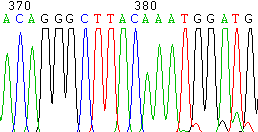


RS

Del


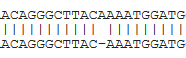


rs134444531 ss2137349058


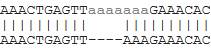

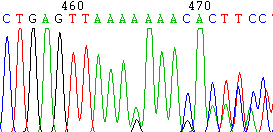


Del

RS


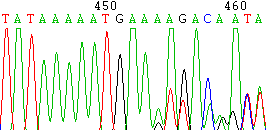

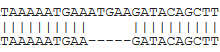


RS

Del

ss2019489562


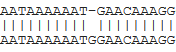

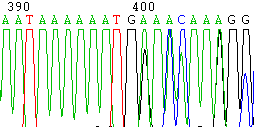


RS

Ins
